# Metropolitan Hospital Sunday Fund

**Published:** 1910-07-30

**Authors:** 


### METROPOLITAN HOSPITAL SUNDAY FUND.

A meeting of the Council of this Fund was held at the Mansion House, on Wednesday, July 27, under the presidency of the Rt. Hon. the Lord Mayor. The minutes of the last ordinary meeting of the Council and the last special meeting were read and approved.

The recommendation of the General Purposes Committee as to the payment of awards to nursing associations was spoken to by Ven. Archdeacon Sinclair, who said a complaint had been received on behalf of certain nursing institutions, and the General Nursing Society, which was at the head of them, received their grants and took off a certain proportion, in a quite open manner, for the working of the central institution. The principle in all the Fund's awards had been that nothing should be subtracted from any of the grants for any such purpose : the whole of the money so well distributed by the Fund was intended to go direct to the institutions which it was proposed to benefit thereby. Therefore, without raising any objection to the methods adopted by the Nursing Institution, the Council would in future prefer to send the grants direct to the institutions concerned.

Sir Wm. Church, K.C.B., proposed :

"That the report of the Committee of Distribution for the year 1910 be and is hereby received, and that the awards thereby recommended be paid as soon as possible, withholding No. 27 pending their giving particulars of Hospital Sunday collections sent them direct, asked for in pursuance of Law IX."

He read Law IX., and said that for many years it had been the habit of the Distribution Committee to send a circular letter to all the ministers who were likely to institute a congregational collection; and, among other places, the circular was sent to the Battersea General Hospital, but no reply had been received therefrom. Therefore the Council suggested that the grant to that particular institution (also known as the Anti-vivisection Hospital) should be withheld until the Council's request had been complied with. In an early letter that Hospital had declined to give the Council any information about its funds separately from the Sunday collections, and as no reply had been received to a second letter, the present suggestion had been put forward.

Dr. Adler (Chief Rabbi) seconded, and expressed deep gratitude to the Distribution Committee for the excellence of their work. The amount awarded this year had nearly equalled that for last year, and the Council was greatly indebted to Mr. Herring for his very munificent bequest. Ministers of religion felt that no part of the work of the Fund was more valuable and acceptable than that which provided for surgical appliances to the very poor.

It was carried.

The following resolution was also agreed to :

"That the thanks of the Council be and are hereby accorded to the Committee of Distribution for the care they have bestowed in the preparation of the awards."

Dr. Flemming moved :

"That the thanks of the Council be and are hereby given to the editors of newspapers who have pleaded the needs of the hospitals and advocated the cause of the Fund."

The Press had, he said, been most helpful; and whenever anything of public benefit was required the newspapers came most generously forward, and not only found space for the purpose, but added their own eloquent appeal to the Council's plea.

The Rev. E. S. Hilliard seconded.

Sir Savill Crossley proposed a cordial vote of thanks to the Rt. Hon. Sir John Knill, Bart., etc., Lord Mayor, who, as President and Treasurer, has devoted his valuable time and influence to the well-being of the Fund.

Sir Henry Burdett seconded the motion, remarking that as the Fund went on year by year, the difficulties in the way of raising the money required would increase. That meant that the President for the time being would be called upon to exercise even greater influence and a larger proportion of his time on behalf of the Fund. The Council was greatly indebted to the present Lord Mayor and to his predecessors for their unfailing help and hospitality on behalf of the Fund.

The resolution was carried by acclamation, and his lordship replied.

The Secretary, Sir Edmund Hay Currie, read a notification that His Majesty the King had graciously consented to become Patron of the Hospital Sunday Fund. (Applause.)

### Sunday Cinematograph Shows.

Dr. Voelcker, writing from Upper Phillimore Gardens, Kensington, asked the Council to dissociate itself from the action of certain hospitals in holding or sanctioning cinematograph displays for the benefit of their funds, and to bring the matter forward so that the Council of the Fund could freely discuss the matter. That should be done before the question was, as it inevitably would be, discussed locally.

A letter had also been received from Prebendary Gurden stating that the shows were held owing to a nominal connection with charity, after deducting expenses. The opening of such shows was not good for any district, and the hospitals should not lend their names to them. The present Fund could put pressure on hospitals for the repression of such displays.

Archdeacon Sinclair said the matter was one of some difficulty and delicacy, and it would be well to refer it to the General Purposes Committee for inquiry and report; it would have been a good thing to do that in the first instance.

Rev. Mr. Pennyfeather seconded the proposal, and spoke on the general question.

The Hon. Sydney Holland (London Hospital) said he was a Sabbatarian in observance, but he did not think it was for the Council of the Fund to tell the people how they should spend their money and what shows they should hold. These particular shows, which he had seen for his own information, contained nothing harmful—a tragedy with a moral, after the style of George R. Sims—and some educational slides, and some slides illustrating a street accident and the subsequent operation at a hospital. The Sunday concerts at Queen's Hall, the Albert Hall, and at the Council band performances, at all of which money was exchanged, were not objected to, and he did not see why the cinematograph shows should be barred, especially as they were not held during hours which interfered with church worship to those who desired to attend church. If that kind of thing were commenced, one did not know where it would stop.

The proposal was carried, and the meeting concluded.

### REPORT OF THE COMMITTEE OF DISTRIBUTION TO THE COUNCIL, 1910.

The Committee of Distribution held its first meeting on Thursday, May 19, and then and at subsequent meetings examined the reports and income and expenditure accounts of the several institutions seeking to participate, and made awards in conformity with Law V.

The executors of the late Mr. George Herring will pay to the treasurer of this Fund on July 28, £13,000, being the balance of income available from the estate, which, with £11,584 received in dividends, etc., upon securities transferred to the Fund, makes the total income from this source to August 1 £24,584.

The committee recommends that this sum be added to the collections, making the total of the Fund to August 1 £63,336. The Fund has not received any legacies this year, against £3,400 last year. This year 256 institutions have applied for grants from the Fund, being two less than in 1909.

The committee, while fully realising the excellent work carried on by the Cancer Hospital, has not made an award this year, for the reason stated in its report of 1906.

The committee now recommends the distribution of £63,000 to the 166 hospitals and institutions, 59 dispensaries and 30 nursing associations shown below.

The committee having reduced the bases of award to 21 institutions, the governing bodies of the same were informed thereof in accordance with Law V. Five deputations attended the committee, and after hearing their several explanations, the awards were finally determined.

Five per cent. of the total sum available for distribution is appropriated to the purchase of surgical appliances during the ensuing year, and 2½ per cent. for district nursing associations.

In compliance with an order of the Council, statistics have been prepared as usual for the special use of its members, showing the number of beds in hospitals, the cost of patients at hospitals and dispensaries, the proportionate expense of management to maintenance, and other valuable information. A copy thereof has been sent to each member of the Council.

John Knill, Lord Mayor, President and Treasurer.

| | |
|---|---|
| John C. Bell. | Robert Grey. |
| F. A. Bevan. | G. H. Makins. |
| William S. Church. | F. H. Norman. |
| Savile Crossley. | E. Parker Young. |
| Joseph C. Dimsdale. | |

Mansion House, E.C.

July 15, 1910.

### Awards Recommended by the Committee of Distribution for the year 1910.

### THIRTY-FOUR GENERAL HOSPITALS.

Charing Cross Hospital, £1,019 13s. 4d.; French Hospital, £340 4s. 2d.; German Hospital, £669 17s. 6d.; Great Northern Central Hospital, £1,140 8s. 4d.; Guy's Hospital, £1,588 18s. 4d.; Hampstead General Hospital, £473 8s. 4d.; North-West London Hospital, £181 2s. 6d.; Italian Hospital, £221 7s. 6d.; Kensington General Hospital, £113 1s. 8d.; King's College Hospital, £1,543 17s. 6d.; London Hospital, £5,941 13s. 4d.; London Homœopathic Hospital, £411 2s. 6d.; Phillips' Memorial Homœopathic Hospital, £47 18s. 4d.; London Temperance Hospital, £653 11s. 8d.; Metropolitan Hospital, £885 10s.; Mildmay Hospital, £244 7s. 6d.; Miller Hospital and Royal Kent Dispensary, £246 5s. 10s.; Poplar Hospital, £616 4s. 2d.; Prince of Wales' General Hospital, Tottenham, £816 10s.; Royal Free Hospital, £1,405 17s. 6s.; St. George's Hospital, £1,545 15s. 10d.; SS. John and Elizabeth Hospital, £99 13s. 4d.; St. John's Hospital, Lewisham, £207; St. Mary's Hospital, £2,267 8s. 4d.; St. Thomas's Hospital, £543 7s. 6d.; Seamen's Hospital Society, £1,630 2s. 6d.; The Battersea General Hospital, £78 11s. 8d.; The Middlesex Hospital and Convalescent Home, £2,523 5s. 10d.; University College Hospital, £2,234 16s. 8d.; Walthamstow, etc. Hospital, £127 9s. 2d.; Wandsworth, Bolingbroke Hospital, £326 15s. 10d.; West Ham Hospital, £505 0s. 10d.; West London Hospital, £1,291 16s. 8d.; Westminster Hospital, £1,243 18s. 4d.

### SPECIAL HOSPITALS.

Six Chest Hospitals.—City of London Hospital for Diseases of the Chest, Victoria Park, £1,103 0s. 10d.; Hospital for Consumption, Brompton, £2,911 8s. 4d.; Mount Vernon Consumption Hospital, Hampstead, £1,395 6s. 8d.; Royal Hospital for Diseases of the Chest, City Road, £547 4s. 2d.; Royal National Sanatorium (Bournemouth), £95 16s. 8d.; Royal National Hospital for Consumption (Ventnor), £239 11s. 8d.

Twenty Children's Hospitals.—Alexandra Hospital for Hip Disease, £309 10s. 10d.; Banstead Surgical Home, £39 5s. 10d.; Barnet Home Hospital, £45 0s. 10d.; Belgrave Hospital for Children, £179 4s. 2d.; Cheyne Hospital for Incurable Children, £77 12s. 6d.; East London Hospital for Children, Shadwell, £857 14s. 2d.; Evelina Hospital for Sick Children, Southwark, £34 10s.; Home for Incurable Children, Hampstead, £44 1s. 8d.; Home for Sick Children, Sydenham, £127 9s. 2d.; Hospital for Sick Children, Gt. Ormond Street, £1,122 4s. 2d.; Infants' Hospital, Westminster, £179 4s. 2d.; Kensington, for Children, and Dispensary, £98 14s. 2d.; Paddington Green Hospital for Children, £268 6s. 8d.; Queen's Hospital for Children, Hackney Road, £851; Royal Waterloo Hospital for Children and Women, £250 2s. 6d.; St. Mary's Hospital, Plaistow, £254 18s. 4d.; St. Monica's Hospital, Brondesbury, £76 13s. 4d.; Victoria Hospital for Children, Chelsea, £744 12s. 6d.; Victoria Home, Margate, £34 10s.; Hospital for Hip Disease, Sevenoaks, £57 10s.

Eight Lying-in Hospitals.—British Lying-in Hospital, Endell Street, £124 11s. 8d.; City of London Lying-in Hospital, City Road, £200; Clapham Maternity Hospital and Dispensary, £38 6s. 8d.; East End Mothers' Home, £91 0s. 10d.; General Lying-in Hospital, Lambeth, £143 15s.; Plaistow Maternity Hospital, £43 2s. 6d.; Queen Charlotte's Lying-in Hospital, Marylebone Road, £506; Home for Mothers and Babies, Woolwich, £62 5s. 10d.

Five Hospitals for Women.—Chelsea Hospital for Women, £314 6s. 8d.; Hospital for Women, Soho Square, £299 19s. 2d.; Grosvenor Hospital for Women and Children, Vincent Square, £161 19s. 2d.; New Hospital for Women, Euston Road, £345; Samaritan Free Hospital, Marylebone Road, £386 4s. 2d.

### TWENTY-THREE OTHER SPECIAL HOSPITALS.

Cancer Hospital, Brompton, nil.; Middlesex Hospital, Cancer Wing, £225 4s. 2d.; London Fever Hospital, Islington, £287 10s.; Gordon Hospital for Fistula, Vauxhall Bridge Road, £30 13s. 4d.; St. Mark's Hospital for Fistula, City Road, £278 17s. 6d.; National Hospital for the Diseases of the Heart, Soho Square, £157 3s. 4d.; Female Lock Hospital, Harrow Road, £201 5s.; Hospital for Epilepsy, Paralysis and other Diseases of the Nervous System, Maida Vale, £241 10s.; National Hospital for the Paralysed and Epileptic, £890 5s. 10d.; West End Hospital for Diseases of the Nervous System, £554 17s. 6d.; Central London Ophthalmic Hospital, Gray's Inn Road, £158 2s. 6d.; Royal Eye Hospital, St. George's Circus, £308 11s. 8d.; Royal London Ophthalmic Hospital, City Road, £978 9s. 2d.; Royal Westminster Ophthalmic Hospital, Charing Cross, £113 1s. 8d.; Western Ophthalmic Hospital, Marylebone Road, £112 2s. 6d.; Royal National

Orthopædic Hospital, Great Portland Street, £399 12s. 6d.; Royal Sea Bathing Hospital, Margate, £172 10s.; St. John's Hospital for Diseases of the Skin, £71 17s. 6d.; St. Peter's Hospital for Stone, Covent Garden, £126 10s.; Central London Throat and Ear Hospital, Gray's Inn Road, £9 11s. 8d.; London Throat Hospital, Great Portland Street, £22 0s. 10d.; Royal Ear Hospital, Dean Street, £40 5s.; Royal Dental Hospital of London, £305 14s. 2d.; National Dental Hospital, 149 Great Portland Street, £52 14s. 2d.

### THIRTY-FOUR CONVALESCENT HOSPITALS.

Metropolitan Convalescent Institution, Walton, £383 6s. 8d.; Metropolitan Convalescent Institution, Broadstairs, £249 3s. 4d.; Metropolitan Convalescent Institution, Bexhill, £444 13s. 4d.; All Saints' Convalescent Hospital, Eastbourne, £383 6s. 8d.; All Saints' Convalescent Home, St. Leonards-on-Sea, £23 19s. 2d.; Ascot Priory Convalescent Home, £81 9s. 2d.; Brentwood Convalescent Home for Children, £10 10s. 10d.; Charing Cross Hospital Convalescent Home, Limpsfield, £58 9s. 2d.; Chelsea Hospital for Women Convalescent Home, St. Leonards, £46; Metropolitan Hospital Home, Cranbrook, £9 11s. 8d.; Deptford Medical Mission Convalescent Home, Bexhill, £17 5s.; Mrs. Gladstone's Convalescent Home, Mitcham, £76 13s. 4d.; Friendly Societies' Convalescent Home, Dover, £95 16s. 8d.; Hahnemann Convalescent Home, Bournemouth, £28 15s.; Hanwell Convalescent Home, £12 9s. 2d.; Hastings, Fairlight Convalescent Home, £28 15s.; Hendon, Ossulston Home, £59 8s. 4d.; Herbert Convalescent Home, Bournemouth, £19 3s. 4d.; Herne Bay Baldwin-Brown Convalescent Home, £57 10s.; Homœopathic Hospital Convalescent Home, Eastbourne, £9 11s. 8d.; Hemel Hempstead Convalescent Home, £19 3s. 4d.; Mrs. Kitto's Convalescent Home, Reigate, £47 18s. 4d.; London Hospital Convalescent Home, Tankerton, £68 0s. 10d.; Mary Wardell Convalescent Home for Scarlet Fever, £57 10s.; Police Seaside Home, Brighton, £51 15s.; St. Andrew's Convalescent Home, Clewer, £95 16s. 8d.; St. Andrew's Convalescent Home, Folkestone, £172 10s.; St. John's Home for Convalescent and Crippled Children, Brighton, £33 10s. 10d.; St. Joseph's Convalescent Home, Bournemouth, £43 2s. 6d.; St. Leonards-on-Sea Convalescent Home for Poor Children, £95 16s. 8d.; St. Mary's Convalescent Home, Broadstairs, £143 15s.; St. Mary's Convalescent Home, Shortlands, £21 1s. 8d.; St. Michael's Convalescent Home, Westgate-on-Sea, £33 10s. 10d.; Seaside Convalescent Hospital, Seaford, £115.

### TWENTY-FOUR COTTAGE HOSPITALS.

Acton Cottage Hospital, £96 15s. 10d.; Beckenham Cottage Hospital, £76 13s. 4d.; Blackheath and Charlton Cottage Hospital, £76 13s. 4d.; Bromley, Kent, Cottage Hospital, £125 10s. 10d.; Bushey Heath Cottage Hospital, £39 5s. 10d.; Canning Town Cottage Hospital, £90 1s. 8d.; Chislehurst, Sidcup and Cray Valley Cottage Hospital, £69; Coldash Cottage Hospital, £21 1s. 8d.; Ealing, £82 8s. 4d.; East Ham Cottage Hospital, £68 0s. 10d.; Eltham Cottage Hospital, £71 17s. 6d.; Enfield Cottage Hospital, £76 13s. 4d.; Epsom and Ewell Cottage Hospital, £43 2s. 6d.; Hounslow Cottage Hospital, £43 2s. 6d.; Livingstone Dartford Cottage Hospital, £91 0s. 10d.; Kingston, Victoria Hospital, £83 7s. 6d.; Mildmay Cottage Hospital, £48 17s. 6d.; Reigate and Redhill Cottage Hospital, £109 5s.; Sidcup Cottage Hospital, £33 10s. 10d.; Tilbury (Passmore Edwards) Cottage Hospital, £47 18s. 4d.; Willesden Cottage Hospital, £105 8s. 4d.; Wimbledon Cottage Hospital, £61 6s. 8d.; Wimbledon (South) Cottage Hospital, £50 15s. 10d.; Wood Green Cottage Hospital, £72 16s. 8d.

### TWELVE INSTITUTIONS.

Hospital for Invalid Gentlewomen, Harley Street, £92 19s. 2d.; St. Saviour's Hospital and Nursing Home, £95 16s. 8d.; Invalid Asylum, Stoke Newington, £31 12s. 6d.; Firs Home, Bournemouth, £36 8s. 4d.; St. Catherine's Home, Ventnor, £19 3s. 4d.; Friedenheim Hospital for the Dying, £299; Free Home for Dying, Clapham, £112 2s. 6d.; St. Luke's House, Pembridge Square, £154 5s. 10d.; Santa Claus Home, Highgate, £59 8s. 4d.; Royal Mineral Water Hospital, Bath, £57 10s.; Winifred House, Holloway, £59 8s. 4d.; All Saints, Highgate, £29 14s. 2d.

### FIFTY-NINE DISPENSARIES.

Battersea Provident Dispensary, £194 10s. 10d.; Billingsgate Dispensary, £55 11s. 8d.; Blackfriars Provident Dispensary, £18 4s. 2d.; Bloomsbury Provident Dispensary, £15 6s. 8d.; Brixton, etc., Dispensary, £46 19s. 2d.; Brompton Provident Dispensary, £20 2s. 6d.; Buxton Street Dispensary, £15 6s. 8d.; Camberwell Provident Dispensary, £75 14s. 2d.; Camden Town Provident Dispensary, £14 7s. 6d.; Chelsea, Brompton and Belgrave Dispensary, £38 6s. 8d.; Chelsea Provident Dispensary, £13 8s. 4d.; Childs Hill Provident Dispensary, £12 9s. 2d.; City Dispensary, £57 10s.; Clapham General and Provident Dispensary, £24 18s. 4d.; Deptford Medical Mission, £23; Eastern Dispensary, £43 2s. 6d.; East Dulwich Provident Dispensary, £53 13s. 4d.; Farringdon General Dispensary, £46; Finsbury Dispensary, £46 19s. 2d.; Forest Hill Provident Dispensary, £35 9s. 2d.; Greenwich Provident Dispensary, £30 13s. 4d.; Hackney Provident Dispensary, £21 1s. 8d.; Hampstead Provident Dispensary, £46 19s. 2d.; Holloway and North Islington Dispensary, £9 11s. 8d.; Islington Dispensary, £53 13s. 4d.; Islington Medical Mission, £50 15s. 10d.; Kennington and Vauxhall Provident Dispensary, £13 8s. 4d.; Kensal Town Provident Dispensary, £11 10s.; Kentish Town Medical Mission, £21 1s. 8d.; Kilburn, Maida Vale and St. John's Wood Dispensary, £35 9s. 2d.; Kilburn Provident Medical Institution, £47 18s. 4d.; London Dispensary, Spitalfields, £15 6s. 8d.; London Medical Mission, Endell Street, £104 9s. 2d.; Margaret Street, for Consumption, £31 12s. 6d.; Metropolitan Dispensary, £60 7s. 6d.; Mildmay Medical Mission Dispensary, £12 9s. 2d.; Notting Hill Provident Dispensary, £18 4s. 2d.; Paddington Provident Dispensary, £34 10s.; Public Dispensary, Drury Lane, W.C., £34 10s.; Queen Adelaide's Dispensary, £24 18s. 4d.; Royal General Dispensary, £28 15s.; Royal Pimlico Provident Dispensary, £39 5s. 10d.; Royal South London Dispensary, £44 1s. 8d.; St. George's, Hanover Square, Dispensary, £31 12s. 6d.; St. John's Wood Provident Dispensary, £47 18s. 4d.; St. Marylebone General Dispensary, £47 18s. 4d.; St. Pancras and Northern Dispensary, £35 9s. 2d.; South Lambeth, Stockwell, and North Brixton Dispensary, £31 12s. 6d.; Stamford Hill, Stoke Newington Dispensary, £47 18s. 4d.; Tower Hamlets Dispensary, £43 2s. 6d.; Walworth Provident Dispensary, £13 8s. 4d.; Wandsworth Common Provident Dispensary, £12 9s. 2d.; Westbourne Provident Dispensary, £18 4s. 2d.; Western Dispensary, £76 13s. 4d.; Western General Dispensary, £91 0s. 10d.; West Ham Provident Dispensary, £11 10s.; Westminster General Dispensary, £46; Whitechapel Provident Dispensary, £33 10s. 10d.; Woolwich Provident Dispensary, £40 5s.

THIRTY NURSING ASSOCIATIONS.

Belvedere, Abbey Wood, £7 11s. 6d.; Brixton, £30 6s.; Central St. Pancras, £22 14s. 6d.; Chelsea and Pimlico, £22 14s. 6d.; Hackney, £22 14s. 6d.; Hammersmith, £53 0s. 6d.; Hampstead, £22 14s. 6d.; Isleworth, £15 3s.; Kensington, £53 0s. 6d.; Kilburn, £7 11s. 6d.; Kingston, £30 6s.; Metropolitan (Bloomsbury), £22 14s. 6d.; St. Olave's (Bermondsey), £30 6s.; Paddington and Marylebone, £37 17s. 6d.; Peckham, £15 3s.; Plaistow, £136 7s.; Plaistow (Maternity), £174 4s. 6d.; Rotherhithe, £15 3s.; Shoreditch, £45 9s.; Sick Room Helps Society, £22 14s. 6d.; Silvertown, £22 14s. 6d.; South London (Battersea), £53 0s. 6d.; Southwark, £37 17s. 6d.; South Wimbledon, £45 9s.; Tottenham, £7 11s. 6d.; Westminster, £30 6s.; Woolwich, £30 6s.; East London, £189 7s. 6d.; North London, £60 12s.; London District, £310 11s. 6d..

